# Floral nectary, nectar production dynamics and chemical composition in five nocturnal *Oenothera* species (Onagraceae) in relation to floral visitors

**DOI:** 10.1007/s00425-017-2748-y

**Published:** 2017-08-04

**Authors:** Sebastian Antoń, Elwira Komoń-Janczara, Bożena Denisow

**Affiliations:** 1 Department of Botany, University of Life Sciences in Lublin, Akademicka 15, 20-950 Lublin, Poland; 2 Department of Biotechnology, Human Nutrition and Science of Food Commodities, University of Life Sciences in Lublin, Skromna 8, 20-704 Lublin, Poland

**Keywords:** Amino acids, Anatomy, Anthesis, Morphology, Pollination syndrome, Protein

## Abstract

***Main conclusion*** **The floral nectars were sucrose-dominant; however, nectar protein and amino acid contents differed, indicating that composition of nitrogenous compounds may vary considerably even between closely related plant species, irrespectively of nectary structure.**

Numerous zoophilous plants attract their pollinators by offering floral nectar; an aqueous solution produced by specialized secretory tissues, known as floral nectaries. Although many papers on nectaries and nectar already exist, there has been a little research into the structure of nectaries and/or nectar production and composition in species belonging to the same genus. To redress this imbalance, we sought, in the present paper, to describe the floral nectary, nectar production, and nectar composition in five nocturnal *Oenothera* species with respect to their floral visitors. The structure of nectaries was similar for all the species investigated, and comprised the epidermis (with nectarostomata), numerous layers of nectary parenchyma, and subsecretory parenchyma. Anthesis for a single flower was short (ca. 10–12 h), and flowers lasted only one night. The release of floral nectar commenced at the bud stage (approx. 4 h before anthesis) and nectar was available to pollinators until petal closure. Nectar concentration was relatively low (ca. 27%) and the nectar was sucrose-dominant, and composed mainly of sucrose, glucose and fructose. The protein content of the nectar was also relatively low (on average, 0.31 µg ml^−1^). Nevertheless, a great variety of amino acids, including both protein and non-protein types, was detected in the nectar profile of the investigated taxa. We noted both diurnal and nocturnal generalist, opportunistic floral insect visitors.

## Introduction

The ecological importance of floral nectar as a food-reward, offered by animal-pollinated plants to their pollen vectors, has long been recognized (Simpson and Neff 1981; Nicolson 2007). Floral nectar is synthesized and produced by floral nectaries, i.e., secretory structures that may be found on different parts of the flower. These vary considerably both in terms of anatomical structure and nectar-secretory mechanisms (Nepi 2007). Nectar is the main floral food-reward for pollinators, and, as such, is subject to selection pressures imposed by nectar consumers. As a consequence, nectar characters can be similar between unrelated plants sharing the same pollination syndrome, or completely different, even between closely related plant species having different pollinators (Baker and Baker 1982).

In general, carbohydrates dominate the total solutes present in floral nectar (Nicolson and Thornburg 2007); however, other compounds, such as amino acids, proteins, and lipids, have also been detected in floral nectars (Baker and Baker 1983a; Heil 2011; Nepi 2014). Moreover, secondary compounds (such as phenols, alkaloids, and terpenoids) that are mostly associated with resistance to herbivory have been documented in floral nectars, and hence, many plants produce nectar that is toxic or repellent to some visitors (Adler 2000). Nectar composition may be conservative due to phylogenetic constraints (Galetto et al. 1998); however, some nectar traits may be subject to ecological factors imposed by the habitat (Stiles and Freeman 1993; Petanidou 2005). For example, nectar production and concentration may be subject to considerable fluctuations resulting from subtle changes in the environment (e.g., temperature, humidity, and wind), as well as other extrinsic factors such as pollinator behaviour, presence of nectar robbers, or nectar contamination by yeasts (Baker and Baker 1983a; Galetto and Bernardello 2004; Herrera et al. 2009). In many cases, nectar components, and consequently, nectar sugar ratios, reflect the type of pollinator, and flower and inflorescence morphology may provide a valuable indicator of the pollination syndrome employed (Baker and Baker 1983b). For example, sucrose-rich nectar has often been recorded for flowers pollinated by hummingbirds or by insects with long mouthparts (e.g., long-tongued bees and butterflies), whereas hexose-rich nectars occur in flowers pollinated by short-tongued bees, flies, and bats (Percival 1961; Baker and Baker 1983b; Stiles and Freeman 1993; Perret et al. 2001; Nicolson 2007). Other carbohydrates, e.g., mannose, arabinose, maltose, stachyose, or xylose, have also been detected in small quantities in floral nectars (Nicolson and Thornburg 2007).

Nitrogenous compounds, i.e., proteins and amino acids, have also been detected in floral nectars (Nicolson and Thornburg 2007), but to date, their ecological significance and evolutionary significance have received a little attention, and it is only recently that these compounds and their actual functions have been investigated in detail (e.g., Petanidou et al. 2006; Nepi et al. 2012; Nepi 2014; Stpiczyńska et al. 2015; Bertazzini and Forlani 2016). Although the nutritive function of nectar proteins cannot be completely ruled out, at present, it would appear that their main role is related to nectar homeostasis (the maintenance of a constant internal environment) and protection from micro-organisms (Nicolson and Thornburg 2007). Alternatively, like carbohydrates, nectar amino acids also play an important role in the attraction of pollinators. Several studies have shown that nectar amino acid profiles are related to specific pollination syndromes (Escalante-Pérez and Heil 2012 and references therein). Indeed, high amino acid concentrations have been reported for the nectar of flowers adapted to pollination by butterflies and other pollinators which cannot feed on pollen, and hence are strongly dependent on nectar amino acids as a nitrogen source (Baker and Baker 1982; Petanidou et al. 2006). Moreover, specific behavioural responses to amino acids have been observed for insect pollinators. It has been experimentally demonstrated that butterflies and bees, for example, are able to detect the presence of a single amino acid in nectar sugar solutions (Inouye and Waller 1984; Bertazzini et al. 2010) and to show preferences for artificial nectars containing a specific mixture of amino acids (Alm et al. 1990). In nature, the quantities of amino acids in nectar are very variable; however, their composition (i.e., the contribution of a particular amino acid in nectar) shows much less variation (Gijbels et al. 2015). Variability in the amounts of nectar amino acids is strongly influenced by the availability of nitrogen (Gardener and Gillman 2001). In addition, non-protein amino acids are also present in floral nectars and their function is thought to be associated with nectar-mediated plant–pollinator interaction and/or protection from invasion by pathogenic micro-organisms (Nepi 2014 and references therein).

Although many papers have been written on nectar production and nectar chemistry for particular plants and how these traits relate to their putative pollinators (e.g., Petanidou 2005), less research has been done on the nectar secretion patterns and nectar chemistry of species belonging to the same genus or tribe (but see, e.g., Perret et al. 2001; Galetto and Bernardello 2004). Given that nectar secretion and nectar chemistry, in particular the carbohydrate and amino acid composition of nectar, generally deserve more attention (Canto et al. 2007; Nepi et al. 2012; Nepi 2014), a comparative examination of several species from the same genus would be valuable in the context of functional nectar ecology.

Five *Oenothera* species that have differences in the origin, distribution in Europe, and status in Poland (alien, native; sensu Rostański et al. 2010) were chosen to examine their floral nectaries, nectar production dynamics, and nectar components to estimate if there are correlations among these features and to discuss the results in the broader context of plant–pollinator interactions. The genus *Oenothera* L. (evening primroses, Onagraceae), comprising about 145 species, has long been used as a model system for studying evolutionary patterns and processes in flowering plants (Raven 1988). Its species are native to North, Central, and South America. In Poland, about 60 taxa have been reported (Rostański and Tokarska-Guzik 1998; Rostański et al. 2010), and most of these are aliens. *Oenothera* species vary in anthesis (diurnal or nocturnal), habit (annual or perennial), and floral characters, e.g., corolla size, hypanthium length, and the presence of trichomes (Rostański et al. 2010). Some *Oenothera* species are fully self-incompatible and outcrossed (e.g., *O. greggii* or *O. riparia*; Gregory 1964; Krakos et al. 2014), others are self-compatible and autogamous (e.g., *O. sessilis* or *O. spachiana*; Krakos et al. 2014) and others have mixed mating systems involving delayed selfing when flowers are not visited and pollinated by animals (e.g., *O. hookeri*; Gregory 1964). The genus is characterized by extremely high species diversity with disparity in flower morphology, and thus, the flowers are visited and pollinated by various pollinator classes (e.g., Gregory 1964; Krakos et al. 2014). However, some *Oenothera* are considered to be specialized and hawkmoth-pollinated (Raguso et al. 2007; Artz et al. 2010). These taxa occur in primarily or secondarily open habitats such as old fields, disturbed soils in industrial sites, roadsides, stream sides, and sand dunes (Rysiak et al. 2005).

Despite the attractiveness of the flowers of *Oenothera* and their importance as crops or invasives in Europe (Mihulka et al. 2003; Tokhtar and Groshenko 2014; Rostański and Verloove 2015), our current knowledge of the floral ecology of this genus is scant, especially concerning floral nectar physiology and composition. To date, only four taxa have been studied for nectar production (Raguso et al. 2007), and only eleven taxa have been incidentally examined for nectar carbohydrate composition via paper chromatography (Stockhouse 1975). The present work was undertaken to study and compare floral nectaries, nectar production and chemistry in five nocturnal *Oenothera* species and to examine quantitatively and qualitatively how these characteristics may relate to potential pollinators and hence successful plant reproduction. To achieve this, we address the following questions: (1) What is the structure of the floral nectary? (2) What are the dynamics of nectar and nectar sugar production throughout the lifetime of the flower? (3) What is the chemical composition of the nectar? (4) What animals visit the flower?

## Materials and methods

### Study site and plant species

The observations were conducted during the period 2013–2014 at the Botanical Garden of the Maria Curie-Skłodowska University in Lublin, SE Poland (51°15′44″N, 22°30′48″E). Five *Oenothera* species of different geographical distribution and status in Poland were compared (Table 1; sensu Rostański et al. 2010): *O. casimiri* Rostański, *O. flaemingina* Hudziok, *O. nuda* Renner ex Rostański, *O. paradoxa* Hudziok, and *O. rubricaulis* Kleb. Since all *Oenothera* species examined in this study are biennials, each year preceding the recording of observations, plants were established as follows. The seeds, obtained from natural populations in Lublin and Mielec in 2012, were sown onto the light soil substrate at the end of April. Subsequently, seedlings were planted out in September in experimental plots. Each year of study, the plants were grown on loess soil, at pH 6–7, at a site fully exposed to the sun.

**Table 1:** Origin and geographical distribution of five *Oenothera* species in Europe (Rostański et al. 2010 and references therein)

Species	Origin	Distribution in Europe	Status in Poland
*O. casimiri* Rostański	Hybrid (*O. biennis* × *O. rubricaulis*)	Scandinavia, Eastern Europe	Apophyte
*O. flaemingina* Hudziok	Hybrid (*O. rubricaulis* × *O. jueterbogensis*)	Germany, Poland	Antropophyte; rare
*O. nuda* Renner ex Rostański	North America	Germany, Poland, Belgium, France, Portugal, Switzerland	Antropophyte; common
*O. paradoxa* Hudziok	Hybrid originated in Europe	France, Belgium, Germany, Poland; often cultivated for seeds	Antropophyte; common
*O. rubricaulis* Kleb	Europe	Central and Eastern Europe	Apophyte

### Microscopy

In 2014, the structure of the nectary was examined for flowers at the beginning of anthesis (i.e., 20:00–21:00 h). Samples of plant tissues to be used for microscopy were collected from individual flowers (*n* = 10) of different individuals (*n* = 5) of each species studied. The position of the nectary in fresh flowers was determined using an Olympus SZX12 (Tokyo, Japan) stereomicroscope. The structure of the nectary was examined by means of light microscopy and scanning electron microscopy.

Following macroscopic observations, floral nectaries were fixed in 2.5% glutaraldehyde in phosphate buffer (pH 7.4; 0.1 M) for 12 h at 4 °C and washed three times in phosphate buffer. They were then post-fixed in a 1% osmium tetroxide solution for 1.5 h at 0 °C and washed three times in distilled water. Subsequently, the fixed material was dehydrated in a graded ethanol series and infiltrated with LR White Resin (LR White acrylic resin, medium grade, Sigma-Aldrich). Following polymerization at 60 °C, semi-thin sections were cut at 0.6–0.9 µm with a glass knife for light microscopy using a Reichert Ultracut-S ultramicrotome (Leica, Vienna, Austria). For general histology, semi-thin sections were stained with 1% (w/v) 1:1 aqueous methylene blue: azure II (Gahan 1984). The presence of insoluble polysaccharides was tested with the Periodic acid-Schiff’s (PAS) reagent after blocking free aldehyde groups. Sections were examined by means of a Nikon Eclipse E200 (Tokyo, Japan) light microscope.

The semi-thin sections were also examined by means of fluorescence microscopy. To test for the presence of cutinized cell walls, semi-thin sections were stained with auramine O (Sigma-Aldrich; 0.01% w/v in 0.05 m Tris/HCl, pH 7.2) for 15 min (Gahan 1984) and rinsed with distilled water. They were then examined by means of a Nikon Eclipse 90i microscope equipped with a fluorescein isothiocyanate (FITC) filter (EXP. 465–495, DM 505; BA 515–555). In addition, autofluorescence of chlorophyll in plastids was tested for fresh, hand-cut sections of the nectary illuminated with UV light. The observations were conducted using a Nikon 90i fluorescence microscope with a digital camera (Nikon Fi1) and NIS-Elements Br 2 software. In each case, control sections were used.

For scanning electron microscopy, samples of floral tissue were fixed in 2.5% glutaraldehyde in phosphate buffer (pH 7.4; 0.1 M) at a temperature of 4 °C for 12 h. The material was then washed in phosphate buffer and dehydrated through a graded acetone series. Subsequently, the plant material was critical-point dried using liquid CO_2_, sputter coated with gold, and examined at an accelerating voltage of 30 kV with a Tescan/Vega LMU scanning electron microscope (Brno, Czech Republic).

### Nectar characteristics and production

The pattern of nectar production was established in 2013 and 2014 during the peak of flowering for each species of *Oenothera* (i.e., early July). The volume of nectar produced was measured in randomly chosen, unvisited flowers at different stages of development. For this purpose, we excluded potential floral insect visitors by bagging inflorescences (*n* = 20–25 per species, per year) from different individuals (*n* = 15–20 per species, per year) in tulle isolators (mesh size <1 mm), which remained in place until nectar sampling. The rate of nectar accumulation was established on three separate dates for each *Oenothera* species studied throughout the lifespan of the flower. Nectar sampling was performed at 2 h intervals commencing with flowers at bud stage (ca. 5 h before anthesis–15:00 h on the first day) until flower senescence (ca. 9:00 h on the following day). At the appropriate time, flowers were removed from plants and the nectar immediately sampled (all nectar measurements were performed within a half hour of excision). Nectar was collected using the micropipette method (Jabłoński 2002). A single sample contained nectar from 1 to 2 flowers. During each interval of nectar sampling, 3–5 samples were collected, resulting in 194 nectar samples taken in both study years for all species. To calculate the mass of nectar, pipettes laden with collected nectar were reweighed using an Ohaus Scout Pro SPU123 analytical balance (Ohaus Corporation, Pine Brook, NJ, USA). Nectar sugar concentration (% w/w), in each collected sample, was determined using an Abbe refractometer type G (Carl Zeiss AG, Jena, Germany).

### Nectar carbohydrate composition

Nectar carbohydrate composition was quantified for 2013 and 2014. We sampled nectar from 45 to 50 flowers (per species) from different plants (*n* = 15–20 per species, per year). A single sample contained nectar collected from 1 to 3 flowers. As a result, 149 nectar samples were collected in both years for all studied species. Nectar was extracted using 3 mm × 15 mm paper-wicks of Whatman No. 1 (McKenna and Thomson 1988). We sampled nectar from flowers at the beginning of anthesis (ca. 21:00 h). Wicks laden with collected nectar were pinned, air-dried, and stored in envelopes at −24 °C until they were analyzed. Nectar samples were analyzed by high-performance liquid chromatography (HPLC), as follows. Wicks were thawed to room temperature and then placed in 0.6 ml distilled water contained in disposable centrifuge tubes, and spun for 0.5 h at 8000*g* at 15 °C. Following filtration of the sample using a sterile syringe filter (0.45 µm pore size), a 0.3 ml aliquot per sample was analyzed using an HPLC apparatus (Gilson Inc., Middelton, WI, USA) equipped with RI K-2300 refractometric detector (Knauer, Berlin, Germany). The carbohydrates were separated using an Aminex HPX-87H column (300 mm long, 7.8 mm i.d., Bio-Rad, Hercules, CA, USA), coupled with a guard column (30 mm long, 4.6 mm i.d.). Elution of the carbohydrates was carried out using a mobile phase comprising 30 mM sulphuric acid (0.5 ml min^−1^) at a column temperature of 42 °C. Mean quantities of the carbohydrates were calculated from samples and expressed as percentage of total sugars.

### Nectar protein content

To establish the total protein content, floral nectar was collected in 2014 from 30 flowers of different individuals (*n* = 10 per species). The nectar samples were pooled and filtered through syringe filters (0.22 µm pore size), obtaining 4–5 samples for each species. The protein content in the fresh nectar samples was established according to Bradford (1976), using bovine serum albumin as the standard. The absorbance of the samples was measured at 595 nm using NanoDrop 2000C (Thermo Scientific, Waltham, MA, USA). The average total protein content measurements (*n* = 3–5 per species) was calculated for each sample.

### Nectar amino acid identification

Nectar for amino acids analysis was collected in 2014 using a glass micropipette. The nectar was sampled from 50 to 65 unvisited flowers at the beginning of anthesis (ca. 21:00 h) from different individuals (*n* = 10 per species). Precautions (flower emasculation) were taken to minimize nectar contamination by pollen, which can release free amino acids into nectar solution (Gottsberger et al. 1990). Nectar was pooled and five samples were obtained for each species; samples were stored at −24 °C until analyzed. Prior to analysis, the samples were thawed to room temperature. Following extraction with 70% ethanol (1:4) for 45 min at 60 °C, the samples were placed in 2 ml lithium citrate buffer and filtered using a sterile syringe filter (0.22 µm pore size). Amino acid analysis was performed by Amino Acid Analyser 400 (Ingos, Praha, Czech Republic) with ion-exchange chromatography and post-column derivatization of ninhydrin. The amino acid derivates were separated using an Ostion LG FA column (450 mm long, 3.7 mm i.d.). A solvent composed of lithium citrate buffer and 0.3 M LiOH was used as a mobile phase at a flow rate of 0.3 ml min^−1^ and at a column temperature of 74 °C. The identification of all amino acids was performed using a dual-channel photometric detector (excitation 440 nm, detection at 570 nm). Chromatograms were analyzed and compared against standards for identification of individual amino acids. The protein amino acids, standard solutions of hydroxyproline, ornithine, taurine, citruline, L-homoserine, β-alanine, phosphoserine, 3-methylhistidine, α-aminobutyric acid (AABA), and γ-aminobutyric acid (GABA) were used.

### Visitors to flowers

Insect visitors were recorded in 2013 and 2014 for each species studied. Diurnal and nocturnal observations were preformed for 5 different days at 1 h intervals, between 20:00 and 9:00 h (GMT + 2 h). Each census of observations was 10–15 min long. For each observation period, all insect visitors to the particular plant species in a selected area (approx. 1 m^2^ for each species) were recorded, photographed, and/or captured for further identification.

### Stigma receptivity

Prior to nectar sampling, the receptivity of stigmas was checked in the field using the Peroxtesmo Ko. (Merck) method (Dafni and Maues 1998). The receptivity of stigmas was tested in 2013 and 2014 in ten flowers and each stage/per species, i.e., (1) in bud stage (2 days and 1 day before anthesis), (2) at the beginning, and (3) at the end of anthesis.

### Data analysis

Statistica 6.0 (Statsoft) software was used for all data analyses. Standard ANOVA procedures were applied to assess interspecific and intraspecific differences in mean values of each of the analyzed criteria (nectar mass, nectar concentration, and sugar mass) for each species and for each year of study. We analyzed differences in nectar carbohydrate composition and protein content between all studied species. To detect differences between the means, post hoc comparison was made by means of the Tukey’s HSD test. Data are presented as mean values ± SD (standard deviation). The level of statistical significance required to measure differences between the means for all analyses was *P* = 0.05.

## Results

**Fig. 1:**
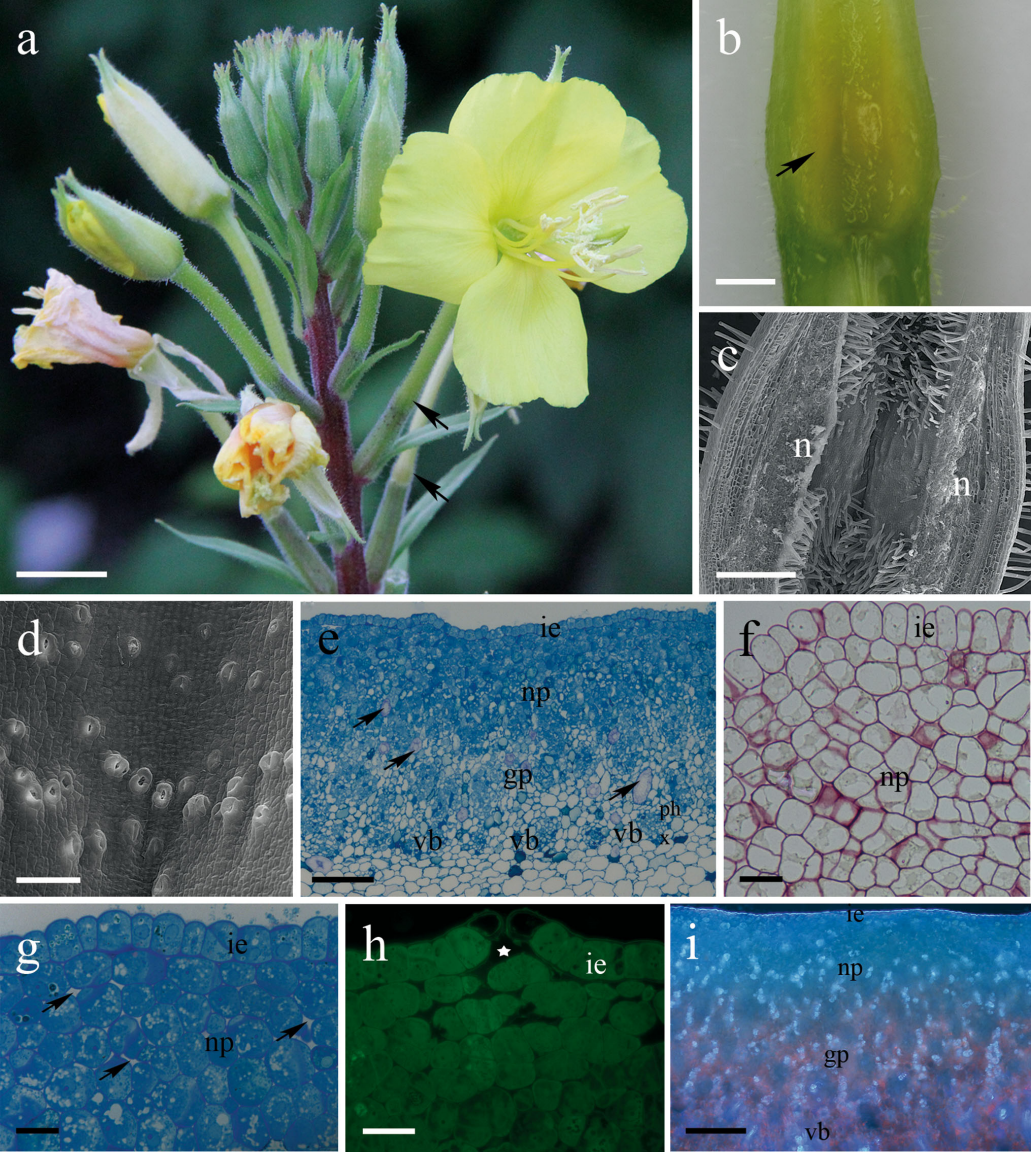
Habit of flower and floral histology of *Oenothera rubricaulis*. **a** Inflorescence with pale yellow flower. **b** Longitudinal section through hypanthium showing floral nectary located basally. **c**, **d** Scanning electron micrographs. **c** Glabrous surface of the internal epidermis of a nectary; note numerous unicellular hairs in the non-secretory area. **d** Numerous nectarostomata on the nectary surface; some stomata occur in pairs. **e**–**i** Light photomicrographs. **e** Transverse section through a hypanthium showing nectary and ground parenchyma with numerous vascular bundles and individual branchysclereids (*arrows*); methylene blue staining. **f** Nectary stained by periodic acid-Schiff’s (PAS); note lack of starch grains in secretory cells. **g** Section of a nectary showing the internal epidermis and nectary parenchyma cells with intensely stained cytoplasm; numerous small intercellular spaces occur between nectary parenchyma cells (*arrows*); methylene blue staining. **h** Nectarostomata with relatively large substomatal space (*asterisk*), auramine O staining. **i** Chlorophyll in the ground parenchyma autofluorescence red. *gp* ground parenchyma; i.e., internal epidermis; *n* nectary, *np* nectary parenchyma, *ph* phloem, *vb* vascular bundles, *x* xylem. *Bars* 1 cm (**a**), 1 mm (**b**), 500 µm (**c**), 100 µm (**d**, **e**, **i**), and 20 µm (**f**, **g**, **h**)

The flowers of the studied *Oenothera* were actinomorphic, scentless to our perception, with four yellow petals (Fig. 1a). The flowers exhibited nocturnal anthesis; the single flower opened in the evening hours (i.e., 20:00–21:00 h) and lasted until early morning (i.e., 7:00–9:00 h). The anthesis of a single flower was relatively short and lasted approx. 10 ± 3 h (mean ± SD calculated across the 2 study years and species).

### Floral nectaries

The morphology and structure of the floral nectary of *Oenothera* species did not differ between the five species investigated, and therefore, in this paper, we present the nectary structure based on that of *O. rubricaulis*.

The nectariferous tissue was located in the basal (proximal) part of the long tubular hypanthium (Fig. 1a–c). Numerous, densely distributed and mature stomata were noted on the surface of the nectary (Fig. 1d). All stomata were raised above the adjacent epidermal cells, and some occurred in pairs (Fig. 1d). The pores or apertures of the modified stomata were flanked by relatively thick cell walls and a distinct substomatal space was present (Fig. 1h). The secretory tissue was composed of a single-layered epidermis, several layers (9–14) of nectary parenchyma cells, and ground parenchyma (Fig. 1e). The cuticle layer covering the outer tangential cell walls of the epidermis was relatively thin and stained only slightly with auramine O (Fig. 1h). The secretory cells of nectary parenchyma were thin-walled and contained intensely staining cytoplasm, a large, centrally located nucleus, plastids, and numerous small vacuoles, whereas the cells of the ground parenchyma were large, highly vacuolated with parietal cytoplasm (Fig. 1g). PAS staining revealed that the nectariferous cells did not accumulate starch neither in nectariferous nor in subsecretory parenchyma (Fig. 1f). The nectary cells were supplied by vascular bundles composed both of phloem and xylem elements that were embedded in the ground parenchyma (Fig. 1e). Numerous small intercellular spaces were also observed between nectary parenchyma cells (Fig. 1f–g). Sclerenchyma cells in the form of individual branchysclereids were frequently seen in the nectary and ground parenchyma (Fig. 1e). Autofluorescence of chlorophyll was observed in the ground parenchyma, but was completely lacking in the internal epidermis and nectariferous parenchyma cells (Fig. 1i).

### Nectar features and production pattern

The release of the floral nectar in all studied *Oenothera* species began at the bud stage, approximately 4–5 h before anthesis. As a result, 35–43% of the maximum volume of nectar produced was secreted early in anthesis (Table 2). The duration of nectar production was similar for all studied species, and lasted ca. 14–18 h. A single flower of each species produced relatively large volumes of nectar (Table 3). The total nectar produced per flower was found to differ significantly between the investigated species (*F*_4,27_ = 7.908, *P* < 0.001; Table 1); however, we found no intraspecific variation in the total quantity of nectar produced per flower over 2 years for any of the species (Table 3).

**Table 2:** Comparison of nectar and nectar production traits among five *Oenothera* species

Trait	*O. casimiri*	*O. flaemingina*	*O. nuda*	*O. paradoxa*	*O. rubricaulis*
Amount at bud stage (prior to anthesis) of the maximum nectar produced by a flower	35%	43%	43%	35%	40%
Secretion period	16 h	14 h	16 h	14 h	18 h
Time of maximum nectar accumulation per flower	7 am (ca. 20 mg)	5 am (ca. 22 mg)	7 am (ca. 19 mg)	5 am (ca. 27 mg)	9 am (ca. 14 mg)
Concentration (% w/w) throughout the anthesis	Decreasing (33–24%)	Constant (ca. 32%)	Constant (ca. 32%)	Decreasing (31-24%)	Decreasing (33-21%)
Resorption period	4 h	6 h	4 h	6 h	4 h

Numeric data represent mean values calculated across 2013 and 2014

**Table 3:** Average nectar production (mg), sugar concentration (% w/w) and sugar amount (mg) in flowers of five *Oenothera* species during a two year study

Species	Year	Nectar production per flower (mg)	Sugar concentration (% w/w)	Sugar amount per flower (mg)
*O. casimiri*	2013	20.4_a_ ± 4.8	22.3_a_ ± 6.4	4.7_a_ ± 2.1
	2014	21.3_a_ ± 3.8	26.2_a_ ± 1.8	5.6_a_ ± 1.2
	Mean for years	20.9_ABC_ ± 3.9	24.6_A_ ± 4.4	5.2_AB_ ± 1.6
*O. flaemingina*	2013	22.2_a_ ± 1.0	28.8_a_ ± 5.2	6.4_a_ ± 1.4
	2014	25.9_a_ ± 9.2	32.0_a_ ± 1.8	8.4_a_ ± 3.6
	Mean for years	24.3_AB_ ± 7.6	30.6_A_ ± 3.7	7.6_A_ ± 2.9
*O. nuda*	2013	17.6_a_ ± 1.7	25.5_a_ ± 1.4	4.3_a_ ± 0.4
	2014	18.5_a_ ± 2.1	32.1_b_ ± 2.1	5.9_a_ ± 0.9
	Mean for years	18.2_AC_ ± 1.8	29.9_A_ ± 3.8	5.4_AB_ ± 1.1
*O. paradoxa*	2013	25.4_a_ ± 2.0	26.8_a_ ± 1.6	6.8_a_ ± 0.5
	2014	28.2_a_ ± 3.6	26.0_a_ ± 0.9	7.3_a_ ± 0.7
	Mean for years	26.8_B_ ± 3.0	26.4_A_ ± 1.2	7.1_A_ ± 0.6
*O. rubricaulis*	2013	13.6_a_ ± 4.7	22.8_a_ ± 2.0	3.1_a_ ± 1.1
	2014	13.9_a_ ± 1.5	32.8_b_ ± 1.0	4.6_a_ ± 0.6
	Mean for years	13.7_C_ ± 3.1	27.8_A_ ± 5.7	3.8_B_ ± 1.1

Data represent mean values ± SD (standard deviation). Means within the columns with the same small letter do not differ significantly between years within species, and means with the same capital letter do not differ significantly between species at *P* < 0.05, based on HSD Tukey test

**Fig. 2:**
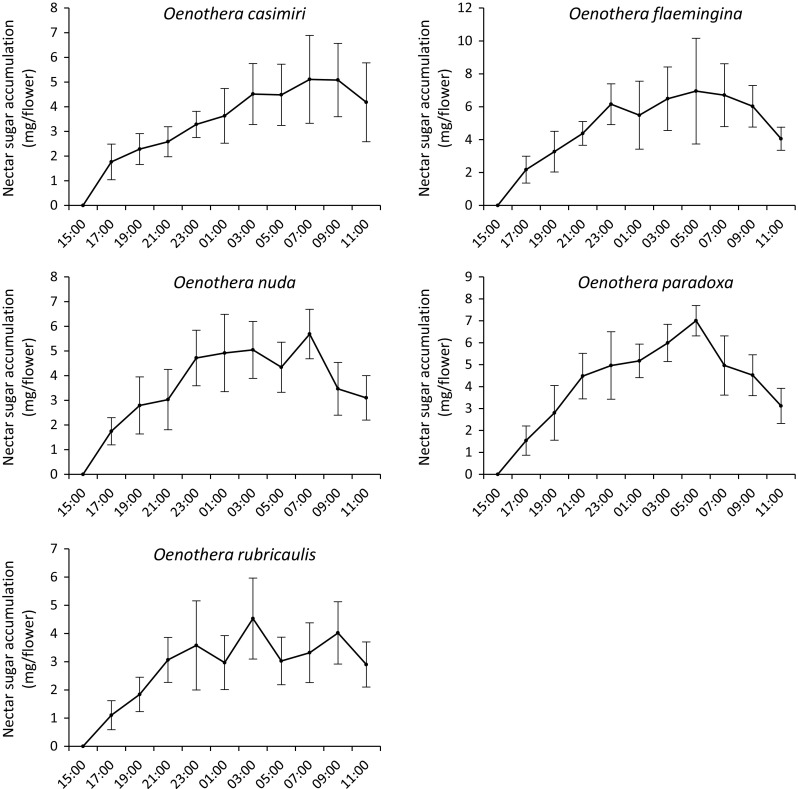
Nectar sugar accumulation per flower (mg) throughout flower lifespan in five *Oenothera* species. Data represent mean values (calculated across 2 study years) ± SD (standard deviation)

Nectar accumulation was gradual throughout the evening and night, reaching its maximum in the early morning hours (Table 2, Fig. 2). The quantity of nectar accumulated was found to differ significantly during the lifespan of the flower, e.g., for *O. casimiri* (*F*_9,57_ = 12.878, *P* < 0.001), *O. flaemingina* (*F*_9,59_ = 9.174, *P* < 0.001), *O. nuda* (*F*_9,43_ = 11.116, *P* < 0.001), *O. paradoxa* (*F*_9,44_ = 20.881, *P* < 0.001), and for *O. rubricaulis* (*F*_9,57_ = 7.813, *P* < 0.001). The mean rate of nectar secretion for the entire secretory period was 1.26 mg/h per flower and 2.52–5.05 mg/h per inflorescence for *O. casimiri*; 1.60 mg/h and 3.20–6.40 mg/h per inflorescence for *O. flaemingina*; 1.16 mg/h per flower and 2.32–4.64 mg/h per inflorescence for *O. nuda*; 1.92 mg/h per flower and 3.84–7.68 mg/h per inflorescence for *O. paradoxa*; and 0.79 mg/h per flower and 1.57–3.15 mg/h per inflorescence for *O. rubricaulis*.

Nectar reabsorption occurred in all *Oenothera* species studied. The reabsorption period lasted for ca. 4–6 h, until abscission of the corolla occurred (Table 2). The mean nectar reabsorption rate was 0.24 mg/h per flower and 0.48–0.96 mg/h per inflorescence for *O. casimiri*; 0.65 mg/h per flower and 1.30–2.61 mg/h per inflorescence for *O. flaemingina*; 2.52 mg/h per flower and 5.04–10.08 mg/h for *O. nuda*; 2.02 mg/h per flower and 4.04–8.08 mg/h per inflorescence for *O. paradoxa*; and 0.56 mg/h per flower and 1.12–2.24 mg/h per inflorescence for *O. rubricaulis*.

### Nectar concentration and carbohydrate composition

Mean nectar sugar concentration for all *Oenothera* species investigated in this study was 27.8 ± 4.4% (mean ± SD, data pooled over two study seasons and plant species). The total nectar concentration did not differ significantly between the investigated species (*F*_4,27_ = 2.572, *P* = 0.06, Table 3). We found no significant differences in the nectar sugar concentration between the study years for *O. casimiri* (*F*_1,5_ = 1.438, *P* = 0.284), *O. flaemingina* (*F*_1,5_ = 1.350, *P* = 0.298), and *O. paradoxa* (*F*_1,4_ = 0.625, *P* = 0.473). However, there was a significant difference in nectar sugar concentration in *O. nuda* (*F*_1,5_ = 15.413, *P* = 0.017) and *O. rubricaulis* (*F*_1,4_ = 58.064, *P* < 0.002) from one year to the next.

Nectar concentration was relatively constant throughout the entire lifespan of flowers of *O. flaemingina* (*F*_9,59_ = 1.220, *P* = 0.301) and *O. nuda* (*F*_9,43_ = 0.501, *P* = 0.865). By contrast, a gradual decline in nectar concentration was observed throughout anthesis for *O. casimiri* (*F*_9,57_ = 5.503, *P* < 0.001), *O. paradoxa* (*F*_9,54_ = 6.689, *P* < 0.001), and *O. rubricaulis* (*F*_9,55_ = 2.430 *P* = 0.021).

The nectar carbohydrates present in all *Oenothera* species were glucose, fructose, and sucrose. No other carbohydrates were detected by HPLC analyses. Nectars were sucrose-dominant in all species, with the proportion of sucrose ranging from 83.3 to 90.1% of the total sugars present, and the sucrose/hexose ratios always exceeding one (Table 4). However, significant differences in nectar carbohydrate composition were found between species for glucose (*F*_4,149_ = 3.290, *P* = 0.013), fructose (*F*_4,149_ = 3.619, *P* = 0.008), sucrose (*F*_4,149_ = 3.862, *P* = 0.005), and sucrose/hexose ratios (*F*_4,149_ = 6.029, *P* < 0.001), but not for hexose ratios (*F*_4,149_ = 0.291, *P* = 0.883; Table 4).

**Table 4:** Percentage carbohydrate composition, sugar ratios (*r*), and hexose ratios (rh) in floral nectar from five *Oenothera* species

Species	Glucose (*G*)	Fructose (*F*)	Sucrose (*S*)	*r* = *S*/(*G* + *F*)	rh = *G*/*F*
*O. casimiri*	7.8_ab_ ± 5.4	7.4_ab_ ± 4.2	84.8_ab_ ± 9.4	7.8_ab_ ± 4.4	1.1_a_ ± 0.5
*O. flaemingina*	4.7_b_ ± 3.5	5.2_b_ ± 2.8	90.1_b_ ± 5.1	10.9_c_ ± 4.5	1.1_a_ ± 0.8
*O. nuda*	8.6_a_ ± 5.3	7.5_ab_ ± 2.5	83.9_a_ ± 7.3	6.5_a_ ± 3.2	1.1_a_ ± 0.5
*O. paradoxa*	8.5_a_ ± 6.4	8.2_a_ ± 4.1	83.3_a_ ± 10.3	6.8_ab_ ± 3.6	0.9_a_ ± 0.4
*O. rubricaulis*	6.1_ab_ ± 4.6	6.2_ab_ ± 3.3	87.7_ab_ ± 7.1	9.8_bc_ ± 5.5	0.9_a_ ± 0.5

Data represent mean values (calculated from samples collected in 2 study years) ± SD (standard deviation). Means within the columns with the same letter do not differ significantly between species at *P* < 0.05, based on HSD Tukey test

### Nectar protein content and amino acid composition

A quantitative estimation of the total protein content of the floral nectar of five *Oenothera* species is shown in Table 5. In general, the protein content of the nectars was relatively low (on average, 0.31 µg/ml, *n* = 26, data pooled over plant species). Significant differences in the total nectar protein content were found to occur between the *Oenothera* species studied (*F*_5,20_ = 142.807, *P* < 0.001). The lowest concentration of proteins was observed for the nectar of *O. nuda* and *O. casimiri* (0.23 µg/ml and 0.25 µg/ml, respectively), whereas the highest concentration of proteins was detected in the nectar of *O. rubricaulis* (0.43 µg/ml).

**Table 5:** Protein content (µg ml^−1^) in the floral nectar from five *Oenothera* species

Species	Protein content (µg ml^−1^)
*O. casimiri*	0.25_a_ ± 0.02
*O. flaemingina*	0.33_b_ ± 0.01
*O. nuda*	0.23_a_ ± 0.02
*O. paradoxa*	0.31_b_ ± 0.01
*O. rubricaulis*	0.43_c_ ± 0.01

Data represent mean values ± SD (standard deviation). Means with the same letters do not differ significantly between species at *P* < 0.05, based on HSD Tukey test

A great variety of amino acids was detected in the floral nectar of each of the species (Table 6). The total quantity of amino acids differed between species, the lowest occurring in the nectar of *O. casimiri* (0.138 µg/mg), and the greatest in the nectar of *O. flaemingina* (0.456 µg/mg). The percentage of each amino acid of the total amino acid concentration also differed between the five plant species. In general, the nectar of each species contained a few, abundant amino acids which exceeded 10% of the total concentration, a number of smaller components with fractions in the range of 5–10%, and a larger number of amino acids with fractions <5% of the total concentration (Table 6).

**Table 6:** Concentration (µg/mg) and relative percentages of amino acids in the floral nectar from five *Oenothera* species

Amino acid	*O. casimiri*		*O. flaemingina*		*O. nuda*		*O. paradoxa*		*O. rubricaulis*	
	Amount (µg/mg)	%	Amount (µg/mg)	%	Amount (µg/mg)	%	Amount (µg/mg)	%	Amount (µg/mg)	%
Alanine	0.008	5.523	0.009	2.126	0.006	3.631	0.011	4.663	0.007	3.041
Cysteine	0.017	12.645	0	0	0	0	0	0	0	0
Aspartic acid	0.006	4.433	0.012	2.630	0	0	0.017	7.037	0.013	5.818
Glutamic acid	0.016	11.483	0.286	62.692	0.083	53.121	0.023	9.580	0.108	47.422
Phenylalanine	0.007	4.942	0.017	3.705	0.009	6.115	0.014	6.147	0.009	4.319
Glycine	0.005	3.634	0.004	0.789	0.003	1.656	0.006	2.755	0.004	1.719
Histidine	0.007	5.233	0.009	2.126	0.002	1.592	0.009	3.815	0.011	4.848
Isoleucine	0.005	3.852	0.012	2.565	0.008	5.353	0.011	4.875	0.007	2.909
Lysine	0	0	0	0	0	0	0.015	6.231	0.002	0.705
Leucine	0.003	2.180	0.012	2.652	0.003	1.971	0.013	5.638	0.008	3.306
Methionine	0	0	0	0	0	0	0.003	1.229	0	0
Arginine	0.008	5.596	0	0	0	0	0.009	4.069	0.005	2.115
Serine	0.008	5.741	0.012	2.565	0.004	2.548	0.017	7.291	0.009	4.054
Threonine	0.002	1.453	0.005	1.162	0.004	2.357	0.006	2.716	0.004	1.719
Valine	0.006	3.997	0.011	2.455	0.008	5.096	0.015	6.147	0.013	5.597
Tyrosine	0.008	5.814	0.010	2.258	0.005	2.866	0.009	3.815	0.007	3.041
*Phosphoserine*	0.022	16.279	0.023	4.954	0.017	10.637	0.029	12.124	0.015	6.611
*Citruline*	0	0	0	0	0	0	0.014	6.019	0	0
α-*aminobutyric acid*	0	0	0.016	3.485	0	0	0	0	0	0
γ-*aminobutyric acid*	0.009	7.195	0.009	1.973	0.005	3.057	0.014	5.849	0.005	2.027
*Ornithine*	0	0	0.002	0.438	0	0	0	0	0.002	0.749
*3*-*methylhistidine*	0	0	0.007	1.425	0	0	0	0	0	0
Total amino acids	0.138		0.456		0.157		0.236		0.223	

Non-protein amino acids are those in italics

Floral nectar contained 11–15 of the 20 protein amino acids. The complete absence of proline in the nectar amino acid profile was characteristic for all species examined here. Glutamic acid was the most abundant amino acid in the nectar of *O. rubricaulis, O. nuda,* and *O. flaemingina* (47, 53, and 63% of the total amino acid concentration, respectively). By contrast, amino acid composition in the nectar of *O. casimiri* and *O. paradoxa* was relatively balanced, the various amino acids present being co-dominant; however, phosphoserine was found to be the most abundant amino acid in the nectar profile of *O. casimiri*. The floral nectar of *Oenothera* species also contained 2–5 non-protein amino acids. Among the most prominent non-protein amino acids (i.e., present in the nectar of all species), were phosphoserine and γ-aminobutyric acid (GABA). Furthermore, the floral nectar of *O. flaemingina* contained five non-protein amino acids in relatively equal concentrations (Table 6).

### Floral insect visitors

During two seasons of observations, we recorded both diurnal and nocturnal insect species visiting flowers of *Oenothera* species (Table 7). They belonged to four taxonomic orders: Coleoptera, Diptera, Hymenoptera, and Lepidoptera. Of the diurnal insects, the most frequent floral visitors were bees of the family Apidae (e.g., *Bombus hortorum, Bombus pascuorum,* and *Bombus terrestris*), whereas most night visits were made by moths of the family Noctuidae (e.g., *Autographa gamma*). The most frequent visitors to flowers of *Oenothera* were nocturnal lepidopterans. This pattern was a general rule and usually the proportion of lepidopteran visits much exceeded 50% in 2013 and 2014; e.g., 59.8 and 71.2% for *O. casimiri*, 76.5 and 68.1% for *O flaemingina*, 61.5 and 58.3% for *O. nuda*, 52.1 and 76.1% for *O. paradoxa,* and 71.7 and 75.7% for *O. rubricaulis*, respectively. Visits by honey bees (*Apis mellifera*) were occasionally observed, as well as by members of Coleoptera and Diptera, and they constituted up to 5% of the visits to the flowers of *Oenothera*.

**Table 7:** Insect floral visitors of five *Oenothera* species in Poland in 2013 and 2014

Species	Total number of insect visits	Coleoptera	Diptera	Hymenoptera	Lepidoptera
*O. casimiri*	195	Scarabaeidae: Oxythyrea funesta*	Syrphidae: *Episyrphus* sp.*	Apidae: Bombus pratorum*, Bombus pascuorum**, Bombus terrestris**, Bombus hortorum**, Apis mellifera*	Noctuidae: Autographa gamma***, Pyrriha umbra*
*O. flaemingina*	184	Scarabaeidae: Oxythyrea funesta*	–	Apidae: Bombus pascuorum*, Bombus hortorum**, Apis mellifera*	Noctuidae: Autographa gamma***
*O. nuda*	169	–	Syrphidae: *Episyrphus* sp.*	Apidae: Bombus terrestris**, Bombus hortorum*	Noctuidae: Autographa gamma***
*O. paradoxa*	171	–	Syrphidae: *Episyrphus* sp.*	Apidae: Bombus terrestris**, Bombus hortorum*, Apis mellifera*	Noctuidae: Autographa gamma***, Pyrriha umbra*
*O. rubricaulis*	179	–	–	Apidae: Bombus lapidarius**, Bombus terrestris**, Apis mellifera*	Noctuidae: Autographa gamma***

Frequency: * rare, ** common, *** very common, – not recorded

### Stigma receptivity

Field tests revealed peroxidase activity in the stigma prior to anthesis in all species studied. Although we observed that the release of nectar commenced 4–5 h before the onset of anthesis, the stigmas were receptive much earlier (approximately 1 day prior to anthesis), and remained receptive until the onset of flower senescence.

## Discussion

### Nectary and nectar production

The floral nectaries of the investigated *Oenothera* species are located at the base of a long hypanthium, where the tube merges with the gynoecium. Microscopy revealed that nectariferous tissue comprises part of this floral tube. The position of the floral nectaries here follows the general pattern found in most members of Onagraceae (Cronquist 1981; Eyde 1981), and seems to be a conservative character for the genus. To our knowledge, however, the literature does not provide any information on the morphology and structure of floral nectaries in other *Oenothera* species.

It is generally agreed that the dynamics of nectar production and volume of nectar produced co-evolved with the requirements and activity of plant pollinators. The *Oenothera* species examined in this study produced relatively large volumes of floral nectar (up to 26.8 mg/flower), while the anthesis of a single flower was short (c.a. 10–12 h) and flowers lasted only one night. Numerous works demonstrate that the intensity of nectar production and the weight of nectar secreted are related to the biomass (thickness and secretory surface) of nectaries and the number of nectarostomata present (e.g., Davis and Gunning 1992; Nepi 2007). In the case of *Oenothera,* floral nectar was synthesized by several layers of epithelial tissue and released via abundant nectarostomata. It is thus reasonable to suppose that the nectary characteristics of *Oenothera* flowers, including nectariferous parenchyma features, are responsible for such large amounts of nectar being produced over such a short period. In fact, a positive correlation has been demonstrated for nectary characters (volume of nectariferous tissue and number of nectarostomata) and the volume of nectar secreted for many plant species (e.g., Konarska 2015, 2017). Interestingly, and in contrast to most floral nectaries (Nepi 2007 and references therein), starch grains were not observed in the nectary of *Oenothera*. In general, the accumulation of starch in the plastids during the period preceding anthesis is characteristical for nectaries producing abundant nectar in a short period of secretory activity, e.g., up to 24 h (Paiva and Machado 2008). This, however, was not the case in *Oenothera*, though their flowers produced relatively high volumes of nectar and the period of nectar secretion and production was shorter than 24 h. Alternatively, quantitative studies have shown that starch accumulation plays only a minor role in nectar production (e.g. Gaffal et al. 2007; Nepi et al. 2011). In the floral nectaries of *Oenothera*, numerous vascular bundles (composed both of phloem and xylem elements) were found in the ground parenchyma. Therefore, nectar sugars are probably uploaded from sieve elements present in the nectary base and then transported as a ‘pre-nectar’ into nectariferous parenchyma. The hypothesis is reinforced by the presence of relatively thin cellulosic cell walls and numerous intercellular spaces between nectary cells, which may facilitate both symplastic and apoplastic routes for transport of the ‘pre-nectar’. Moreover, chloroplasts (revealed by chlorophyll autofluorescence) were observed in the floral nectaries of *Oenothera*, suggesting that nectary cells may be directly involved in production of nectar sugars via an assimilation process. These observations indicate varied nectary supply with carbohydrates and energy required for the production and secretion of floral nectar. The presence of diverse sources of nectar components and their role in flower function and subsequent fruit development has also been demonstrated for the nectaries of other flowers (e.g., Pacini et al. 2003; Antoń and Kamińska 2015).

According to Josens and Farina (1997), the large volume of nectar of relatively low sugar concentration (c.a. 27%) found in *Oenothera* species examined in the present study, together with other floral features, may be related to pollination by hawkmoths. However, we did not observe the presence of hawkmoths in our study during the years of the study. Conversely, based on the classification proposed by Nicolson (2007), the various nectar characteristics (volume, sugar concentration, and nectar carbohydrate composition) of *Oenothera* species studied in the present paper suggest adaptation to a large variety of pollinator classes. This seems to be true at least for the species studied here, as their flowers were visited by generalist insects, and nocturnal Lepidopterans (i.e. *Autographa gamma*) were the main insect visitors. However, the absence of adaptation to any specific pollinator class cannot be excluded.

In most flowering plants, nectar is secreted before pollinators commence their foraging activity and/or before flowers open. In *Oenothera*, the release of floral nectar began at the bud stage (c.a. 4–5 h before flower opening), and therefore, nectar was available continuously from the onset of anthesis (i.e., evening hours) until petal closure (i.e., early morning hours). In our study, a great variety of insects, including both diurnal and nocturnal taxa, visited *Oenothera* species (see Table 7); therefore, the pattern of nectar production can be correlated to potential insect pollinators observed in the field. The large volume of accumulated nectar present at the beginning of anthesis in the evening (i.e., up to 43% of the maximum volume of nectar produced by a single flower) is probably an adaptation to attract nocturnal pollinators, since during this period, stigmas are already receptive (approx. 1 day before anthesis) and pollen is presented for dispersal. Conversely, however, highest accumulative nectar production was observed in the early morning hours, suggesting an adaptation for pollination by diurnal insect visitors, as the stigmas remain receptive until petal closure. This complex pattern of nectar production and accumulation may also be beneficial in other respects. In the investigated *Oenothera*, despite the large number of flowers per inflorescence (c.a. 70–150), only 3–5 flowers were in anthesis at the same time and the amount of nectar offered by a single inflorescence was presumably not sufficient to fulfil the energetic requirements of visiting insects. In addition, variations in nectar availability between flowers on a single plant and between individual plants could perhaps influence insect foraging behaviour. Therefore, diurnal nectar-feeding insects, as well as nocturnal Lepidopterans that feed solely on nectar, need to search for nectar in other *Oenothera* plants, thereby promoting cross pollination. A similar pattern of nectar distribution and its potential influence on pollinator movements has also been proposed for nocturnal and short-living flowers of *Mucuna urens* (Fabaceae) (Agostini et al. 2011).

Shortly, after maximum accumulative nectar production was reached, a reduction in the volume of nectar and nectar sugar concentration was observed for all species studied, strongly suggesting nectar reabsorption. Here, nectar was presented in a long, tubular hypanthium without direct contact with the environment, and therefore, protected from evaporation. Furthermore, if this was to occur, the volume of floral nectar would decrease and its total sugar concentration increase. Nectar reabsorption is a common feature of many flowers and has been demonstrated for numerous plant species, irrespective of the age or sexual expression of the flower (Pacini and Nepi 2007 and references therein). Two main functions of nectar reabsorption have been proposed, i.e., a nectar homeostatic mechanism and the recovery of resources invested in nectar production (Nepi and Stpiczyńska 2008 and references therein). According to Cruden et al. (1983), nectar reabsorption takes place once maximum nectar production is reached and this, in particular, appears to be especially true of *Oenothera.* Although nectar reabsorption, like nectar secretion, requires a considerable expenditure of energy (Nepi and Stpiczyńska 2008 and references therein), flowers that reabsorb nectar may at least reclaim a portion of the resources invested in nectar production. This strategy has been demonstrated or proposed for several plant species (e.g. Búrquez and Corbet 1991; Agostini et al. 2011). According to Southwick (1984), the energy invested in nectar production may amount to twice the energy invested in seed production. For *Oenothera* species which produce a great number of fruits with numerous seeds (Rostański et al. 2010), recovery of resources may, therefore, be advantageous, since constituents of reabsorbed nectar may be reutilized during seed development (Nepi and Stpiczyńska 2008 and references therein). Analogous pattern of nectar and sugar reabsorption has also been documented for *Mucuna urens*, a species with similar nectar secretion and reabsorption periods (Agostini et al. 2011).

### Nectar concentration and carbohydrate composition

Floral nectar chemistry, including nectar carbohydrate composition, may differ significantly between populations, individuals, species or subspecies, or even between flowers on the same plant (e.g., Baker and Baker 1983b; Galetto and Bernardello 2004; Petanidou 2005; Antoń and Denisow 2014; Antoń et al. 2017). According to the classification of Baker and Baker (1983a), the floral nectars of all *Oenothera* species are sucrose-dominant (the sucrose/(glucose + fructose) ratio being always above 6.5). Elaborate sucrose-dominant nectar carbohydrate composition is essentially associated with pollination by long-tongued bees and butterflies (Baker and Baker 1983b). According to the latter authors, the low proportion of glucose and high proportion of sucrose (over 50%), that also characterizes all *Oenothera* species studied, might also be associated with moth syndrome. However, our data on nectar carbohydrate composition did not entirely match the moth preferences proposed by Baker and Baker (1983b), since we also observed short-tongued insects (e.g., *Apis mellifera* and *Bombus terrestris*) feeding on the floral nectar of *Oenothera* spp. According to Galetto and Bernardello (2004), nectar characteristics are not always similar for plants visited by the same animal taxa as was formerly thought. Indeed, hexose-rich nectar was observed in bumblebee-pollinated *Fritillaria meleagris* (Stpiczyńska et al. 2012), and sucrose-rich nectars were noted for both bee- and wasp-pollinated species occurring in the Mediterranean flora (Petanidou 2005). Our findings are in agreement with the previous results for other Onagraceae taxa, including *Oenothera* spp. (Stockhouse 1975; Nicolson and Thornburg 2007), demonstrating that predominance of sucrose in floral nectars occurs irrespective of flower morphology (but see Antoń et al. 2017). Apparently, nectar carbohydrate composition in *Oenothera* is a more conservative feature than is flower morphology, suggesting that phylogenetic constraints may strongly influence nectar chemistry in that genus.

Considering the nectar sugar concentrations of all the *Oenothera* spp. investigated, we observed that the values were all rather low (on average 27.8 ± 4.4%) and thus matched the hawkmoth syndrome well (Baker and Baker 1983b; Stiles and Freeman 1993). According to Baker and Baker (1982), lower sugar concentration means that nectar is less viscous and hence more easily extracted, which appears to be especially important for Lepidoptera feeding through long proboscis. However, the low nectar sugar concentration recorded in our survey contrasts markedly with the actual visitation pattern observed for insects. Besides nocturnal moths, we did also observe short- and long-tongued bees eagerly visiting *Oenothera* spp. and consuming floral nectar. Much of the literature claims that optimal nectar sugar concentrations of bee-pollinated flowers in temperate regions are usually higher (i.e., as much as 40–56%; Nicolson 2007 and references therein) than those recorded in our study for *Oenothera*. In practice, bees collect floral nectar from a much wider range of concentrations (i.e., 15–65%), and the relationship between the preferences of bees and sugar concentration is likewise non-linear (Seeley 1986). It is thus reasonable to suppose that bees play an important role in pollination of these *Oenothera* species due to relatively high frequency of observed visits. However, more observation on foraging behaviour and pollinator efficiency experiments will examine this possibility.

In addition, dilute nectars from the studied species are consistent with measurements from four *Oenothera* species from North America (Raguso et al. 2007). Mitchell and Paton (1990) demonstrated that the nectar concentration preferred by insects can be much lower when the actual sugar intake is measured using a fixed amount of sugar in a variable volume of water. According to the latter authors, even seemingly dilute nectar may be preferred by insect visitors, providing sufficient sugar reward. This is probably also the case for *Oenothera* species, since their flowers offer copious volumes of nectar, and consequently, similar large amounts of sugars. It would appear that by diluting a given quantity of sugar, the nectar produced is able to attract a range of different pollinators and hence limit the possible reduction of fitness by over-production of sugars. Furthermore, nectar containing low concentrations of sugar may deter opportunistic insects preferring more concentrated nectars from visiting the flowers (Bolten and Feinsinger 1978).

### Nectar protein content and amino acid composition

Owing to its great nutritional value, nectar must be protected from opportunistic exploiters, which may be animals (the so-called ‘nectar thieves’) or airborne and/or pollinator-borne micro-organisms (e.g., bacteria and fungi). This protection is provided by the presence of specific substances that may be components of nectar, and these include secondary compounds and proteins (González-Teuber et al. 2010; Escalante-Pérez and Heil 2012). Of the two, proteins appear to be the main constituents involved in nectar protection (Nicolson and Thornburg 2007; Nepi et al. 2011). We found the total protein content of the floral nectar of *Oenothera* species of the present study to be relatively low (on average, 0.31 µg/ml). This is probably related specifically to the way that nectar is presented, namely, within a long, tubular hypanthium that may offer it protection from nectar robbers and from direct exposure to the atmosphere, and thus, from contamination by yeasts and/or bacteria. It would thus appear that nectar proteins in *Oenothera* function mainly as enzymes rather than in nectar protection. Therefore, our findings support the hypothesis that the more ephemeral and concealed the nectar, the less protein it contains (Heil 2011). Furthermore, differences in the total nectar protein content observed between species may also be attributable to diverse dynamics of nectar production and reabsorption. It has also been proposed that differences in the protein content of nectar may also explain various dynamics of nectar production and reabsorption in male and female flowers of *Cucurbita pepo* (Cucurbitaceae) (Nepi et al. 2011).

Although present in much smaller quantities than carbohydrates in floral nectar of *Oenothera* species, amino acids are present at sufficient concentration to provide pollinators with a valuable nitrogen supply (Baker and Baker 1976). A great variety of amino acids, both protein and non-protein types, was detected in the nectar profile of the investigated taxa. Whereas amino acid concentration was variable, 13 amino acids were commonly present in the floral nectar of all these taxa, and these amino acids also occur in the nectar of other flowering plants (e.g., Baker and Baker 1976; Petanidou et al. 2006; Nepi et al. 2012). Eleven (in *O. nuda*) to fifteen (in *O. paradoxa*) of the 20 protein amino acids were found, together with non-protein amino acids. This complex amino acid profile confirms the high nutritive value of *Oenothera* nectar for insects, including honeybees, bumblebees, butterflies, and other insects that visit these flowers.

Our data show that the overall amino acid concentrations differ between species and that some amino acids are more prevalent than others. In *O. flaemingina*, *O. nuda, and O. rubricaulis*, for example, the most abundant amino acid, representing more than 47% of the total amino acid content of nectar is glutamic acid, whereas the nectar of *O. casimiri* and *O. paradoxa* has relatively balanced amino acid concentrations. High levels of the amino acids phosphoserine, histidine, and phenylalanine were also present in the nectar profile. Variability in the amino acid composition of nectar between the investigated *Oenothera* species is not an unusual feature. In fact, nectar amino acid composition may vary between plant species of the same genus, as in *Aquilegia* (*Ranunculaceae*) (Baker and Baker 1976), or may differ slightly, even between flowers of the same species, as in *Caryocar* (Caryocaraceae) (Gottsberger et al. 1984). According to Baker and Baker (1976), nectar amino acid composition, like other adaptive characters, may be used as a taxonomic and phylogenetic indicator.

Although rich in a wide variety of amino acids, the nectar of none of the species studied had an ideal ratio of those essential amino acids required for the complete growth and development of insects, including bees (Dress et al. 1997). Although not essential, glutamic acid is an important proteinogenic amino acid in insect nutrition, as it contributes to the growth of insects and is incorporated from the adult diet into the eggs (Dadd 1973; Mevi-Schutz and Erhardt 2005). Glutamic acid was also found to be one of the major amino acids both in the floral and extrafloral nectar of *Gossypium hirsutum* (Malvaceae) (Gilliam et al. 1983), as well as in the extrafloral nectar of the carnivorous plant *Sarracenia purpurea* (Sarraceniaceae) (Dress et al. 1997). In addition, extreme dominance of a particular amino acid in the floral nectar profile has also been observed for phenylalanine in the case of phryganic nectars (Petanidou et al. 2006).

The present study also reveals significant amounts of particular amino acids in the nectar of five *Oenothera* species, suggesting that their presence here may affect the behaviour of potential pollinators. Phenylalanine, for example, is one of the ten essential amino acids required by honeybees (Chapman 1983) and is particularly important in the attraction of insect pollinators. Indeed, phenylalanine is one of the most common amino acids in honey and is a precursor of a specific fragrance component, namely 2-phenylethanol (Thawley 1969). Moreover, it is phagostimulatory to insects, because it stimulates sugar cell receptors and is frequently found at high concentrations (as much as 50% of the total amino acids) in the floral nectar of plants from Mediterranean regions which are pollinated by long-tongued bees (Petanidou et al. 2006).

Interestingly, γ-aminobutyric acid (GABA), a non-protein amino acid, was present in the floral nectar of all studied *Oenothera* species, and appeared to be particularly important for floral insect visitors. Specifically, it has been reported that GABA, together with phenylalanine, stimulates taste chemoreceptors sensitive to sugars, and thus increases feeding behaviour in some insects (Nepi 2014 and references therein). According to Petanidou et al. (2006), the phagostimulatory effect of GABA may be related to the probable co-presence of NaCl, a salt on which GABA is strongly dependent, and it has been speculated that this combination of GABA-NaCl is the most potent phagostimulant found in the flora of Mediterranean regions. In addition, it has been suggested that extracellular GABA is involved in the communication of plants with other organisms and also accumulates in response to infection by fungi and bacteria (Nepi et al. 2012). For example, accumulated, extracellular GABA was found to reduce the virulence of *Agrobacterium tumefaciens* (Rhizobiaceae) in tobacco leaves (Chevrot et al. 2006). These observations suggest that GABA may provide protection from invasion by pathogenic organisms, a function that is mostly associated with nectar proteins.

In conclusion, our comparative studies indicate that the structure of the floral nectaries of five species of *Oenothera* investigated in this paper is similar, and several other nectary features (i.e., numerous layers of epithelial tissue, various sources of nectar components, and presence of numerous nectarostomata) enable rapid production of large volumes of floral nectar during the course a relatively brief anthesis. The floral nectars of all the *Oenothera* species investigated were sucrose-dominant. We noted significant differences in their nectar protein and amino acid profiles, indicating that the composition and quantities of nitrogenous compounds present in nectar may vary considerably even between closely related plant species, irrespectively of nectary structure. Although sucrose-dominant nectar was presented in a long hypanthium and total concentration amounted ca. 27% (on average), both diurnal (including short and long-tongued bees) and nocturnal generalist insect visitors were observed. However, the studied species do not differ appreciably in the diversity or abundance of visitors attracted to the flowers. Since nectar is not easily accessible in tubular flowers, actual sugar intake and nectar composition are just as important in determining plant–visitor relations as is floral morphology. More research is needed to improve our understanding of floral rewards in *Oenothera*, both for elucidating its pollination ecology and its breeding system.

#### *Author contribution statement*

BD and SA conceived the general strategy; SA performed the experiments; EKJ and SA performed biochemical analysis; SA and BD interpreted the results; SA wrote the manuscript.

## Abbreviation

GABA: γ-Aminobutyric acid
